# Low rate of latent autoimmune diabetes in adults (LADA) in patients followed for type 2 diabetes: A single center’s experience in Turkey

**DOI:** 10.20945/2359-3997000000268

**Published:** 2020-06-19

**Authors:** Abbas Ali Tam, Didem Ozdemir, Nagihan Bestepe, Fatma Dilek Dellal, Muhammet Cuneyt Bilginer, Sevgul Faki, Cemile Bicer, Reyhan Ersoy, Bekir Cakir

**Affiliations:** 1 Department of Endocrinology and Metabolism Yildirim Beyazit University Faculty of Medicine Ankara Turkey Department of Endocrinology and Metabolism, Yildirim Beyazit University Faculty of Medicine, Ankara, Turkey; 2 Department of Biochemistry Yildirim Beyazit University Faculty of Medicine Ankara Turkey Department of Biochemistry, Yildirim Beyazit University Faculty of Medicine, Ankara, Turkey

**Keywords:** Type 2 diabetes, latent autoimmune diabetes in adults, HbA1c, c-peptide, body mass index, Turkey

## Abstract

**Objective:**

In this study, we aimed to determine the frequency of and the clinical and metabolic features of patients with latent autoimmune diabetes in adults (LADA) at a single center in Turkey.

**Subjects and methods:**

Patients over 30 years of age diagnosed with type 2 diabetes who did not require insulin for a minimum of 6 months following diagnosis were included. Data from 324 patients (163 women; 161 men), with a mean age of 54.97 ± 7.53 years, were analyzed in the study. Levels of antibodies to glutamate decarboxylase (anti-GAD) were measured in all patients, and LADA was diagnosed in patients testing positive for anti-GAD antibodies.

**Results:**

Anti-GAD positivity was identified in 5 patients (1.5%). Family history of diabetes, body mass index (BMI), age, sex distribution, insulin resistance, serum triglycerides, high-density lipoprotein, and low-density lipoprotein were similar in the LADA and type 2 diabetes patients. Median HbA1c was significantly higher (10.8% vs. 7.38%, p = 0.002) and fasting C-peptide was lower (0.75 ng/mL vs. 2.82 ng/mL, p = 0.009) in patients with LADA compared to in those with type 2 diabetes. Among the 5 patients with LADA, 4 were positive for antithyroid peroxidase antibodies. The median disease duration was relatively shorter among patients with LADA (4 years vs. 7 years, p = 0.105).

**Conclusion:**

We observed a LADA frequency of 1.5% among Turkish patients followed for type 2 diabetes. The presence of obesity and metabolic syndrome did not exclude LADA, and patients with LADA had worse glycemic control than patients with type 2 diabetes did.

## INTRODUCTION

Latent autoimmune diabetes in adults (LADA) is a form of autoimmune diabetes affecting adult patients that is marked by circulating autoantibodies against β-cells. These patients do not require insulin upon diagnosis ( 1 ). The biochemical, genetic, and phenotypic characteristics of both type 1 and type 2 diabetes can be observed in this heterogeneous group of disease ( 2 , 3 ). At the time of a diabetes diagnosis, it is impossible to differentiate LADA from phenotypic type 2 diabetes unless islet autoantibodies are measured ( 4 ).

Most patients with LADA are misdiagnosed and treated as having type 2 diabetes due to the disease’s latent nature, indistinguishable symptoms, and laboratory results. In these patients, blood glucose regulation deteriorates within a few months or years, which requires insulin treatment. Early diagnosis and treatment with insulin might delay the disease’s progression ( 5 ). In addition, patients with LADA have worse glycemic regulation than patients with type 2 diabetes ( 3 ). Although not shown in all studies, lower prevalence of metabolic syndrome and of cardiometabolic risk factors such as obesity, hypertension, and dyslipidemia have been reported in LADA patients compared to in type 2 diabetics ( 6 - 8 ).

Epidemiological studies suggest that LADA may account for 2-12% of all cases of diabetes ( 9 ). In a study of 6,156 patients in Europe, the prevalence of LADA was 9.7% ( 6 ). Among 4,880 adults with type 2 diabetes, 5.9% were positive for autoantibodies in the multicenter LADA China Study ( 10 ). To our knowledge, only 1 study has been conducted on LADA in Turkey, which reported a 31% prevalence of LADA among 54 patients ( 11 ). The universal guidelines for subjecting adult diabetics at high risk of LADA to islet antibody testing are poorly defined. Such testing is important because the treatment options may differ for LADA versus type 2 diabetes ( 12 ). In this study, we aimed to determine the frequency of LADA among Turkish patients who were diagnosed with type 2 diabetes and to evaluate the clinical and metabolic features of LADA patients.

## SUBJECTS AND METHODS

This cross-sectional study was performed between 2014 and 2017 in the Endocrinology and Metabolism outpatient clinic of Ankara Yildirim Beyazit University. Type 2 diabetes patients who were diagnosed after age 30 and did not require insulin during the first 6 months following diagnosis were included. Exogenous insulin reduces hyperglycemic stress, decreases the severity of insulitis, and supports β-cell function. To avoid possible bias in relation to insulin use and to compose a more unique study group, we did not include patients who were on insulin treatment at the time of recruitment ( 13 ). Patients with type 1 diabetes, gestational diabetes, or secondary diabetes were also excluded. LADA was defined according to the Immunology of Diabetes Society (IDS) criteria: the presence of glutamic acid decarboxylase antibodies (anti-GAD), a lack of requirement for insulin treatment for at least 6 months, and over 30 years of age at the time of diagnosis ( 14 ). Patients positive for anti-GAD were accepted as having LADA, and those negative for anti-GAD were accepted as having type 2 diabetes. Anti-GAD measurement was not repeated in patients with positive results. Demographic, biochemical, clinical, and medication (antihypertensive, antihyperlipidemic, and antidiabetic) data were recorded, and anthropometric and biochemical measurements were performed. All patients were asked about diabetes history among first-degree relatives. Body mass index (BMI) was calculated as weight in kilograms divided by the square of height in meters (kg/m^2^). The patients were categorized as underweight, normal, overweight, or obese when their BMI was < 18.5 kg/m^2^, 18.5-24.9 kg/m^2^, 25-29.9 kg/m^2^, and ≥ 30 kg/m^2^, respectively. Waist circumference was measured between the lower rib margin and the anterior superior iliac spine, and the hip circumference was measured over the maximum of the buttock ( 15 ).

Blood samples were taken between 8:00 AM and 10:00 AM after a minimum 8-10 hours of fasting. Glucose was measured with the hexokinase method using a Cobas c501 (Roche). The immunoturbimetric method, using a Cobas 6000 (Roche), was used for HbA1c analysis. Insulin and C-peptide were analyzed by Cobas e 601 electrochemiluminescence immunoassay (Roche). Anti-GAD was measured with the immunoradiometric method, using the Beckman Coulter assay (Fullerton, CA, USA) and an LB 211 Gamma Counter (Berthold). The cut-off value for anti-GAD was 1 IU/mL, and values above 1 IU/mL were considered positive (reference range: 0-1 IU/mL). The method’s analytical sensitivity was 0.2 IU/mL, and the intra-assay and inter-assay precision CV were 3.7% and 6.9%, respectively. Only anti-GAD65 autoantibodies were detected by the test, according to the test’s principles.

Serum C-peptide levels were used to indicate β-cell capacity. Insulin resistance was estimated with homeostasis model assessment (HOMA-IR): {[fasting glucose (mg/dL) × fasting insulin (µU/mL)] / 405} ( 16 ). Poor glycemic control was defined as HbA1c ≥ 7% ( 3 ).

Thyroid autoantibodies were measured by chemiluminescence methods (Immulite 2000, Diagnostic Products Corp., Los Angeles, CA, USA, and UniCel DXI 800, Beckman Coulter, Brea, CA, USA). The normal levels for anti-TPO and anti-Tg were 0-35 IU/mL and 0-40 IU/mL, respectively. Thyroid antibody levels over the upper range were accepted as positive.

### Statistical analysis

The distributions of metric variables such as age and BMI were examined by Shapiro-Wilk’s tests and normality plots. The age of the whole sample was summarized as mean ± standard deviation. All continuous variables were presented as medians (IQR [interquartile range], 1^st^ quartile to 3^rd^ quartile) because of their nonnormal distribution in groups. Numbers and percentages (%) were given for the categorical variables. Mann-Whitney *U* test and chi-square were used to compare metrics and categorical variables, respectively. p < 0.05 was considered statistically significant. All statistical analyses were performed via IBM SPSS Statistics 21.0 (IBM Corp., Armonk, NY).

## RESULTS

The data of 324 patients were analyzed, with a mean age of 54.97 ± 7.53 years (median: 55; range: 35-70). Of these, 161 were male (49.7%), and 163 (50.3%) were female. Five (1.5%) were positive, while 319 (98.5%) were negative, for anti-GAD antibodies. The anti-GAD titers in positive patients were 5.1, 25.7, 26.19, 658.0, and 2000.0 IU/mL (median: 26.19, IQR: 15.4-1329.0). No significant differences existed between patients with LADA and those with type 2 diabetes with respect to age, gender distribution, median BMI, hip and waist circumference, or hip-to-waist ratio ( Table 1 ). The ages of the patients with LADA were 35, 43, 51, 52, and 56. Three of the 5 patients (60.0%) with LADA and 163 of the 319 patients (54.5%) with type 2 diabetes were obese. The median duration of diabetes was 4 years (IQR: 1.5-7.5) among LADA patients and 7 years (IQR: 4-10) among type 2 diabetes patients (p = 0.105). With the exception of 1 patient with LADA who had suffered from the disease for 10 years, all of the other LADA patients had the disease for 5 years, at most.

**Table 1:** Demographical and anthropometric features of patients with LADA and type 2 diabetes

	LADA (n = 5) Median (IQR) n (%)	Type 2 diabetes (n = 319) Median (IQR) n (%)	p
Age (years)	51 (39-54)	55 (50-60)	0.053
Gender			
Female	0 (0.0)	161 (50.5)	0.061
Male	5 (100.0)	158 (49.5)	
BMI (kg/m^2^)	30.8 (24.8-37.7)	30.5 (27.5-34.0)^1^	0.975
Thin-normal	1 (20.0)	31 (10.4)	
Overweight	1 (20.0)	105 (35.1)	
Obese	3 (60.0)	163 (54.5)	
Hip circumference (cm)	109.0 (97.0-122.5)	108.0 (101.0-115.8)^2^	0.955
Waist circumference (cm)	90.0 (84.0-112.0)	103.0 (97.0-111.3)^3^	0.109
Hip/waist ratio	1.149 (1.047-1.244)	1.052 (0.991-1.108)^2^	0.060
Disease duration (years)	4 (1.5-7.5)	7 (4-10)^4^	0.105
≤ 5 years	4 (80.0)	120 (38.5)	
Family history	3 (60.0)	202 (65.0)	1.000

^1^: n = 299, ^2^: n = 296; ^3^: n = 298; ^4^: n = 312

LADA: latent autoimmune diabetes in adults; BMI: body mass index; IQR: interquartile range (1^st^ quartile-3^rd^ quartile).

The patients with LADA had significantly higher HbA1c and lower insulin and C-peptide levels than those with type 2 diabetes did. Four of the 5 patients (80.0%) with LADA and 11.9% of the type 2 diabetes patients tested positive for thyroid peroxidase antibodies (anti-TPO) (p = 0.001). LADA and type 2 diabetes patients did not differ in fasting blood glucose, HOMA-IR, thyrotropin (TSH), free triiodothyronine (fT3), free thyroxine (fT4), thyroglobulin antibody (anti-Tg) positivity, or lipid parameters. The median number of oral antidiabetics was significantly higher among patients with LADA (p = 0.036; Table 2 ).

**Table 2:** Laboratory findings and medications of patients with LADA and type 2 diabetes

	LADA (n = 5) Median (IQR) n (%)	Type 2 diabetes (n = 319) Median (IQR) n (%)	p
FBG (mg/dL)	213.0 (120.5-316.0)	141.0 (122.0-177.0)	0.111
HbA1c (%)	10.80 (9.44-12.85)	7.38 (6.61-8.70)^1^	0.002
Insulin ( μU/mL)	5.43 (3.09-9.39)	10.08 (7.01-14.40)	0.033
HOMA-IR	1.938 (1.211-5.362)	3.655 (2.371-5.967)	0.193
C peptide (ng/mL)	0.75 (0.21-2.40)	2.82 (2.20-3.55)	0.009
LDL (mg/dL)	107.6 (94.4-156.3)	117.0 (92.0-135.8)^2^	0.856
HDL(mg/dL)	66.0 (36.0-91.5)	44.0 (37.0-51.8)^2^	0.149
TG (mg/dL)	65.0 (57.5-179.0)	149.5 (112.0-222.0)^2^	0.075
TSH (uIU/mL)	1.71 (0.90-2.41)	1.95 (1.32-2.68)^3^	0.428
fT4 (ng/dL)	1.43 (1.22-1.55)	1.31 (1.19-1.41)^4^	0.297
fT3 (pg/mL)	2.81 (2.43-3.03)	3.07 (2.80-3.34)^5^	0.111
Anti TPOAb positivity	4 (80.0)	37 (11.9)	0.001
Anti TgAb positivity	1 (20.0)	22 (7.1)	0.317
Diabetic medicine	3 (2-3)	2 (0-4)	0.036
Antihypertensive drug	2 (40.0)	70 (21.9)	0.308

^1^: n = 318, ^2^: n = 240; ^3^: n = 297; ^4^: n = 298; ^5^: n = 296

LADA: latent autoimmune diabetes in adults; IQR: interquartile range (1^st^ quartile-3^rd^ quartile); FBG: fasting blood glucose; HDL: high-density lipoprotein; LDL: low-density lipoprotein; TG: triglyceride; TSH: thyrotrophin; fT4: free thyroxine; fT3: free triiodothyronine; Anti TPOAb: anti-thyroid peroxidase antibody; Anti TgAb: anti-thyroglobulin antibody.

## DISCUSSION

In this study, we found a 1.5% proportion of LADA among our patients diagnosed with type 2 diabetes. Ethnic origin is one of the main factors that affects the prevalence of LADA in different studies. In Europe, the highest rates were reported in people of Northern European origins (7-14%) ( 1 ). In India, autoantibody positivity was reported as 44.6%, which was the highest frequency ( 17 ), while in Alaska, no patients with type 2 diabetes were found to have anti-GAD antibodies in a study of 264 patients ( 18 ). The frequency of LADA in our study was similar to those of studies from Malaysia, United Arab Emirates, and South Korea, which reported a prevalence of 2.9%, 2.6%, and 1.7%, respectively ( 19 - 21 ). Wide variations in the prevalence of LADA worldwide may be due to differences in methods, study design, or inclusion criteria, such as ethnicity, age at diagnosis, length of the insulin-free period, number and type of autoantibodies tested, and the sensitivity and specificity of autoantibody assays.

To our knowledge, the single study from Turkey included 54 patients and found a considerably higher rate of LADA (31%) than the present study did. However, the sample size was quite small, and patients who were positive for anti-GAD antibodies were defined to have LADA without other diagnostic criteria being considered ( 11 ). The authors did not present any data regarding insulin use within the first 6 months of diagnosis, and the lower limit of age as an inclusion criterion was not defined. These methodological differences between that study and ours make it difficult to compare the 2 studies. In addition, the prevalence of LADA might differ in different regions of the same country. One such example is the lower prevalence of LADA in northern compared to southern Spain (10.9-14.7% *vs.* 5.6-7.2%) ( 22 ). Similarly, different prevalence rates were reported in northern and southern regions of Italy ( 23 ) and China ( 10 ).

Although LADA and type 1 diabetes have some genetic and immunological similarities, their differences in antibody, T-cell, and some genetic features suggest that the underlying autoimmune disease processes might differ ( 24 ). The development of autoimmunity in patients with LADA could arise as a consequence of either chronic inflammatory responses such as obesity or of a more specific environmental trigger that can promote the activation of an autoimmune process similar to that which occurs in type 1 diabetes. Wilkin was the first to hypothesize that obesity and weight gain had important roles in inducing beta cell apoptosis. According to the accelerator hypothesis, weight gain represents an overlay between type 1 and type 2 diabetes. Weight gain causes increased insulin resistance and worsens hyperglycaemia, which further contributes to the immunogenic destruction of β cells due to glucotoxicity ( 25 ).

One trend is to measure islet cell antibodies in patients with type 2 diabetes when clinical suspicion of LADA is high, which is generally based on BMI. Type 2 diabetics with normal weight are considered candidates for LADA, whereas those who are overweight or obese are considered protected from LADA; therefore, antibody measurement is typically neglected for the latter. However, this approach is not convincing since overweight or obese LADA patients have previously been reported ( 5 ). With the increasing prevalence of obesity worldwide, it is increasingly difficult to distinguish LADA from type 2 diabetes based on BMI alone ( 1 , 5 ). In our study, over half of patients with both type 2 diabetes and LADA were obese, demonstrating that the presence of obesity does not exclude LADA.

Previous studies have reported that metabolic syndrome can be seen in patients with LADA, but at a lower prevalence than in patients with type 2 diabetes ( 26 ). In a recent study, metabolic syndrome was found in 88.8% of patients with type 2 diabetes and 41.9% of patients with LADA ( 27 ). Also, metabolic syndrome was not more prevalent in patients with autoimmune diabetes than in control subjects, suggesting that metabolic syndrome is not a characteristic of autoimmune diabetes ( 27 ). We found a higher mean HDL cholesterol level and lower mean triglyceride and LDL cholesterol levels in patients with LADA compared to in patients with type 2 diabetes; however, the differences were not statistically significant. In addition, BMI, waist and hip circumferences, and family history of diabetes were similar among both groups of patients. These data suggest that patients with LADA and type 2 diabetes share the same risk factors and that the presence of metabolic syndrome does not necessarily exclude LADA. However, the small sample size of LADA patients in our study decreases the statistical power of our findings.

Studies have shown that β-cell dysfunction is higher among patients with LADA than among type 2 patients but lower than among type 1 diabetics ( 27 ). Gottsater and cols. ( 28 ) found that insulin levels, fasting, and stimulated C-peptide secretion in LADA patients were in between those of type 1 and type 2 diabetic patients. In addition, the decline in C-peptide levels over time was faster among LADA patients than among type 2 diabetics. This decline in β-cell function is the main cause for why LADA patients require insulin ( 29 ). Although these patients are more likely to use insulin, optimal glucose regulation is generally more difficult than for those with type 2 diabetes ( 8 ). In a study by Andersen and cols. ( 3 ) during a follow-up period of 107 months (8.9 years), patients with LADA had a higher prevalence of poor glycemic control than patients with type 2 diabetes did (67.8% *vs.* 53%). In another study by Mollo and cols. ( 30 ) LADA was associated with higher deterioration in β-cell function and worse glycemic control (HbA1c of 8.1% *vs.* 7.6%) than type 2 diabetes was. In our study, patients with LADA had lower fasting C-peptide and higher HbA1c levels. In addition, these patients required a higher frequency and dose of medications for optimal glucose regulation. These findings indicated that residual β-cell function in patients with LADA was worse than in patients with type 2 diabetes.

Patients with LADA have increased risk of thyroid and adrenal antibody positivity and celiac disease ( 29 ). Although the association between type 1 diabetes and autoimmune thyroid diseases is well established, little data exists about the risk of thyroid autoimmunity in patients with LADA ( 31 ). Some studies have shown increased rates of anti-TPO among patients with LADA compared to among patients with type 2 diabetes ( 31 ). The Ehime study reported that 49.2% of patients with LADA were positive for anti-TPO ( 32 ). In the study by Szepietowska and cols. ( 33 ) anti-TPO positivity was found in 38.6% of patients. In our study, 4 of the 5 patients with LADA were positive for anti-TPO, suggesting a more active autoimmune process. Maioli and cols. ( 34 ) reported that while high anti-GAD titer was not a major risk factor for disease progression in LADA patients, anti-TPO positivity was an independent risk factor. In our study, glycemic regulation was poor in LADA patients, and we thought that high anti-TPO positivity might have contributed to this result.

LADA has also been associated with other autoimmune diseases. Zampetti and cols. ( 35 ) observed autoantibodies such as anti-TPO, anti-21-hydroxylase, anti-tissue transglutaminase, and antiparietal antibodies at higher frequencies in patients with high anti-GAD titers. Thus, the authors suggested routine screening of other organ-specific antibodies in patients with high anti-GAD titers.

Although autoimmune diseases are more prevalent in women, all of the LADA patients were male in our study. While epidemiological studies have shown controversial results, some studies have reported a higher prevalence of LADA in men ( 12 , 36 ). This might be related to demographic features of the population and their genetic predisposition.

To the best of our knowledge, this is the largest study to date to evaluate patients with LADA in Turkey. However, our study has some limitations. Firstly, this was a hospital-based study, and selection of the study population may have had some bias. Our center is a referral center, and patients with poor glycemic control constitute a considerable proportion of diabetic patients. Secondly, the median disease duration was 4 years in patients positive for anti-GAD, while it was longer in anti-GAD-negative patients. Anti-GAD titers fluctuate and decrease over time, which may have caused lower-than-actual frequency of LADA. As another limitation, we considered only anti-GAD positivity for the diagnosis of LADA. Although anti-GAD is by far the most common and most sensitive autoantibody and previous studies have reported that measuring other islet autoantibodies seems to add little value to anti-GAD in diagnosing LADA ( 1 , 6 - 8 ), we cannot exclude the possibility that analysis of other antibodies (e.g., islet antigen-2 antibody, insulin autoantibody, and zinc transporter 8) could contribute to the results, particularly among those with negative anti-GAD. Exogenous insulin would support β-cell function, thus reducing hyperglycemic stress and decreasing the severity of insulitis. Insulin treatment for LADA patients is not strictly defined and largely depends on the physician’s clinical experience and the patient’s desire. Considering these biases, to construct a more homogeneous/unique study population, we did not include patients on insulin treatment at the time of recruitment. This might have also contributed to the low frequency of LADA in our study.

Although our patient group was not small, only 5 patients were diagnosed with LADA, which prevents us from generalizing our results. The prevalence of diabetes in our country was reported as 7.2% in the TURDEP-I study from 1997 ( 37 ). This prevalence increased to 16.5% in the TURDEP-II study, which was performed 12 years later ( 38 ). Thus, the prevalence of diabetes appears to be increasing, with 6.5 million current adult diabetics in Turkey. Despite the low frequency of LADA in our study (1.5%), the high prevalence of diabetes in Turkey can render the results meaningful. Identifying LADA patients early can provide optimal treatment for these patients and the preservation of their β-cell functions. Otherwise, suboptimal glucose regulation might lead to increased long‐term diabetes complications.

In conclusion, LADA should be suspected in patients with recent onset (< 5 years) of poorly regulated diabetes, low C-peptide levels, and positive thyroid autoimmunity independent of BMI. The presence of obesity and metabolic syndrome does not exclude LADA. Due to the small sample size of the LADA group, the results of the current study cannot be generalized. Further epidemiological and clinical studies with larger sample sizes will demonstrate the prevalence and increase awareness of LADA.
